# Effect of magnesium sulfate and personalised dietary guidance on hemodynamics and inflammatory cytokines in pregnancy-induced hypertension

**DOI:** 10.5937/jomb0-57165

**Published:** 2025-09-05

**Authors:** Miao Shen, Ying Zhuang, Yaning Zheng, Mengqin Wang, Jiexun Wang, Shuoying Lyu

**Affiliations:** 1 Nanjing Drum Tower Hospital, Affiliated Hospital of Medical School, Nanjing University, Nanjing, Jiangsu, 210008, China

**Keywords:** magnesium sulfate, pregnancy-induced hypertension, personalised dietary guidance, inflammatory cytokines, hemodynamics, magnezijum-sulfat, hipertenzija izazvana trudnoćom, personalizovane dijetetske smernice, inflamatorni citokini, hemodinamika

## Abstract

**Background:**

Pregnancy-induced hypertension (PIH) is a significant cause of maternal and neonatal complications, often linked to vascular dysfunction and inflammatory responses. This study aimed to evaluate the effects of magnesium sulfate (MS) combined with personalised dietary guidance on hemodynamic parameters and inflammatory cytokine profiles in PIH patients.

**Methods:**

A total of 108 PIH patients were randomly assigned to two groups: a research group (MS and dietary guidance) and a control group (dietary guidance only). Hemodynamic parameters, including systolic and diastolic blood pressure (SBP, DBP), plasma viscosity (PV), and erythrocyte aggregation index (EI), were measured, along with inflammatory cytokines [Interleukin-6 (IL-6), Interleukin-10 (IL-10), and Interleukin-1b (IL-1b)], before and after treatment.

**Results:**

The research group, which received both MS and dietary guidance, showed significant reductions in SBP, DBP, PV, and EI compared to the control group. Additionally, inflammatory cytokines IL-6 and IL-1b were significantly reduced in the research group, indicating an improvement in the inflammatory response. While IL-10 levels decreased in both groups, this change was not statistically significant.

**Conclusions:**

Combining magnesium sulfate and personalised dietary guidance effectively improves hemodynamic stability and reduces inflammatory markers in PIH patients.

## Abstract (sr)

**Uvod:**

Hipertenzija izazvana trudnoćom (HIT) predstavlja značajan uzrok komplikacija kod majki i novorođenih beba koje su često povezane sa vaskularnom disfunkcijom i inflamatornim odgovorima. Ova studija imala je za cilj da proceni efekte magnezijum-sulfata (MS) u kombinaciji sa personalizovanim dijetetskim smernicama na hemodinamske parametre i profile inflamatornih citokina kod pacijentkinja sa HIT-om.

**Metode:**

Ukupno 108 pacijentkinja sa HIT-om je nasumično raspoređeno u dve grupe: grupu ispitanika (MS i dijetetske smernice) i kontrolnu grupu (samo dijetetske smernice). Mereni su hemodinamski parametri, uključujući sistolni i dijastolni krvni pritisak (SKP, DKP), viskoznost plazme (VP) i indeks agregacije eritrocita (IAE), kao i inflamatorni citokini [interleukin-6 (IL-6), interleukin-10 (IL-10) i interleukin-1b (IL-1b)], pre i nakon tretmana.

**Rezultati:**

Grupa ispitanika, koja je primala i MS i dijetetske smernice, pokazala je značajno smanjenje SKP, DKP, VP i IAE u poređenju sa kontrolnom grupom. Pored toga, inflamatorni citokini IL-6 i IL-1b bili su značajno redukovani u grupi ispitanika, što ukazuje na poboljšanje inflamatornog odgovora. Iako su nivoi IL-10 opali u obe grupe, ova promena nije bila statistički značajna.

**Zaključak:**

Kombinacija magnezijum-sulfata i personalizovanih dijetetskih smernica efikasno poboljšava hemodinamsku stabilnost i smanjuje inflamatorne markere kod pacijentkinja sa HIT-om.

## Introduction

Pregnancy-induced hypertension (PIH) refers to a condition in which pregnancy and hypertension coexist, making it one of the most common diseases in obstetrics. The pathogenesis of PIH remains unclear; however, it is believed to be influenced by various factors such as heredity, excessive inflammatory and immune responses during pregnancy, uteroplacental ischemia, and malnutrition [1]. The global prevalence of PIH in pregnant women ranges from 5 to 12 per cent, with more than 30% of these cases resulting in adverse delivery outcomes [2]. Therefore, the prevention and management of PIH are of utmost importance to safeguard pregnant women’s and newborns’ health. Currently, PIH is treated with medications such as magnesium sulfate (MS), which helps lower blood pressure (BP) and alleviate vasospasms [3] [4]. MS has demonstrated its effectiveness in reducing maternal complications associated with PIH. However, concerns persist regarding its potential impact on fetal hemodynamics, as it can cross the placenta and affect the fetus, potentially leading to congenital malformations and increasing the risk of stillbirth [5].

At the same time, we cannot ignore the importance of the inflammatory response in PIH. A key component of the inflammatory response in PIH is the dysregulation of cytokines, particularly interleukins such as Interleukin-6 (IL-6), Interleukin-10 (IL-10), and Interleukin-1β (IL-1β) [6]. IL-6 is a proinflammatory cytokine shown to elevate during PIH and contribute to vascular dysfunction, while IL-10, an anti-inflammatory cytokine, may be downregulated in this condition [7]. Additionally, IL-1β, another pro-inflammatory cytokine, plays a significant role in endothelial dysfunction and developing vascular spasms associated with PIH [8]. These inflammatory cytokines exacerbate the underlying pathology of PIH, leading to complications such as urinary protein increase, oedema, multi-organ damage, intrauterine fetal growth restriction, distress, and stillbirth [3]. Although MS has shown promise in controlling the blood pressure response to PIH [9], its role concerning inflammatory markers, including IL-6, IL-10, and IL-1β, remains underexplored.

Therefore, this study aims to investigate the combined effects of MS and personalised dietary guidance on the hemodynamics and inflammatory responses in PIH patients, mainly focusing on the changes in IL-6, IL-10, and IL-1β levels. By examining these biomarkers, we hope to provide new insights into the pathophysiology of PIH and offer alternative strategies for improving maternal and fetal outcomes in the future.

## Materials and methods

### Sample size calculation

The sample size required for the study was calculated using GPower software with effect size=0.5, α=0.05, and power=0.95, which showed that a minimum of 42 study subjects were required in each group.

### Study population

One hundred and eight PIH patients admitted to our hospital from April 2022 to December 2023 were selected by random sampling, who were then divided into a research group (n=54) for MS combined with personalised dietary guidance and a control group (n=54) for personalised dietary guidance using a random number, none of the study participants were aware of their grouping. This study was approved by the hospital’s Ethics Committee and was carried out strictly with the Helsinki Declaration; all study subjects signed an informed consent form. The two groups’ age, gravidity, gestational weeks, etc., were compared, and no statistical significance was found (P>0.05, Table 1).

**Table 1:** Comparison of clinical data.

Groups (n=54)	Age	Week of pregnancy	Weight (kg)	Body mass index<br>(kg/m^2^)	History of gynaecological<br>diseases yes/no
Control	29.54±2.13	38.85±2.37	59.48±2.88	22.59±1.99	34 (62.96)/20 (37.04)
Research	28.96±2.87	38.31±3.03	59.31±3.26	23.05±2.05	29 (53.70)/25 (46.30)
t/χ^2^	1.180	1.026	0.282	1.184	0.952
P	0.241	0.307	0.779	0.239	0.329

### Eligibility and exclusion criteria

Inclusion criteria: (1) The patients met the diagnostic criteria for PIH [10], with systolic blood pressure (SBP) 140 mmHg and/or diastolic blood pressure (DBP) 90 mmHg, elevated aspartate amino transferase (AST) or alanine aminotransferase (ALT), or platelet count < 100×10^9^/L, accompanied by varying degrees of dyspnea, chest tightness, and other symptoms of cardiac insufficiency. (2) The patients were informed about this study and signed informed consent forms. (3) The patients’ medical records were complete. Exclusion criteria: Patients with (1) acute infectious and connective diseases, (2) chronic renal insufficiency [11], (3) systemic organic heart disease before pregnancy, (4) hypertension diagnosed before pregnancy, or (5) communication disorders (individuals have difficulty expressing their thoughts and feelings clearly or understanding the intentions and intents of others in verbal, written, or non-verbal communication), were excluded.

### Methods

Personalised dietary guidance: Dietary guidelines were set by our dietitian, and a handbook was made and distributed to each patient. The patient’s diet is explained face-to-face on the first day of admission, thanks to a nurse from the Department of Obstetrics and Gynecology who monitors the patient’s diet after admission. They were instructed not to consume pickled or fried foods; instead, a diversified diet was encouraged, with cereals as the main ingredient and reasonable combinations of course and fine grains. For those with severe oedema, the sodium content in daily dishes should not exceed 5 g, and foods with high sodium salt content, such as pickled pickles, thick broth, pickles, canned products, sausages, and sauces, should be avoided. Pregnant women were advised to avoid eating animal fat. They were encouraged to consume milk (cow milk or pasteurised goats’ milk, cheese, yoghurt, etc.), cereals (millet, oats, buckwheat, black and white sesame, sorghum, corn, etc.), legumes (black beans, red beans, green beans, mung beans, etc.), and animal meat (lean pig, cow, sheep). It is recommended to have a daily protein intake of 100 g and a saturated fatty acid calorie intake of less than 10%. The above foods can be consumed 3–4 times a week. The energy intake during pregnancy was advised to increase by 840 kJ/d compared with that during non-pregnancy, with a total of about 9,685 kJ/d. The research group were treated with 25% MS (Shanghai Xudong Haipu Pharmaceutical Co., Ltd., H31020666). 60 mL of MS was added to 100 mL of 5% glucose injection for intravenous drip, which was completed within 1 hour. Then, another 60 mL of MS was added to 500 mL of 5% glucose injection for intravenous infusion for 4 hours. The total therapeutic dose was 16-21 g/d. The personalised dietary guidance continues from the patient’s admission until they are discharged from the hospital.

### Sample collection and testing

BP was measured using a blood pressure (BP) meter (Anke, M8081) before and after treatment (sitting position). In addition, fasting venous blood was collected to assess various biomarkers, including the whole blood viscosity (WBV), plasma viscosity (PV), erythrocyte aggregation index (EI), and inflammatory cytokines such as IL-6, IL-10, and IL-1β. WBV, PV, and EI were analysed using a blood rheology analyser (Kangyu Medical Devices Co., Ltd., HL-5000). Blood samples were processed to extract plasma for cytokine analysis using enzyme-linked immunosorbent assay (ELISA) kits specifically for IL-6, IL-10, and IL-1β; following the manufacturer’s protocols, the kits were purchased from Pujian Bio (Wuhan) Technology Co. The levels of these cytokines were measured to assess the inflammatory status and their potential association with the treatment outcomes in both groups.

### Endpoints

BP (SBP and DBP) and hemodynamics (WBV, PV, and EI) were measured in both groups before and after treatment. The assessment of psychological status used the Hamilton Depression Scale (HAMD) and Hamilton Anxiety Scale (HAMA) [12], with higher scores indicating more serious negative emotions. Adverse reactions and delivery outcomes were also analysed. Furthermore, the pregnant women were investigated for satisfaction (very satisfied, satisfied, in need of improvement, and dissatisfied) using an anonymous rating survey (10-point scale) at discharge; total satisfaction rate = (very satisfied+satisfied) cases/total number of people ×100% [37726059].

### Statistical analysis

Statistical analysis was performed using SPSS 25.0 (IBM, USA). Chi-square tests were used to compare count data (e.g., pregnancy history, delivery outcomes). For continuous variables such as BP, cytokine levels, and nutritional protein levels, Shapiro-Wilk was used to detect the normal distribution of the data; the independent sample t-test was used for inter-group comparisons, and paired t-tests were used for within-group comparisons before and after treatment. A significance level of P<0.05 was considered statistically significant.

## Results

### Hemodynamics was better in the research group after the treatment

**Figure 1:**
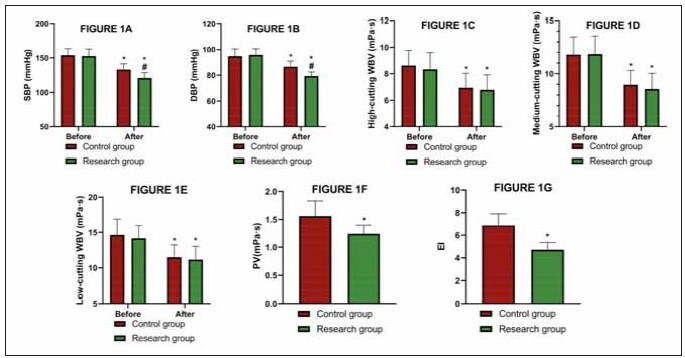
Comparison of A) SBP, B) DBP, C) High-cutting WBV, D) Medium-cutting WBV, E) Low-cutting WBV, F) PV, and G) EI. * denotes P<0.05 compared with before treatment, # denotes P<0.05 compared with control group. Systolic blood pressure, SBP; diastolic blood pressure, DBP; whole blood viscosity, WBV; plasma viscosity, PV; erythrocyte aggregation index, EI.

No marked inter-group differences were found in the hemodynamic test results before treatment (P>0.05). Both groups showed reduced SBP and DBP after treatment, especially in the research group (P<0.05). Meanwhile, the post-treatment high-, moderate-, and low-shear WBV, as well as PV and EI in the research group, were (6.78±1.13) mPa·s, (8.56±1.48) mPa·s, (11.16±1.87) mPa·s, (1.24± 0.16) mPa·s, and (4.72±0.63), respectively, of which the high-, moderate-, and low-shear WBV were similar to those in the control group (P>0.05), and PV and EI were lower (P<0.05; Figure 1).

### Inflammatory factors were lower in the research group after the treatment

**Figure 2:**
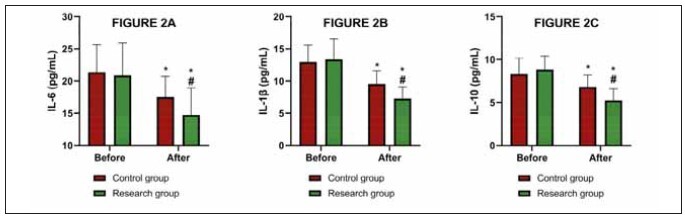
Comparison of A) IL-6, B) IL-1β, and C) IL-10.

The analysis of cytokine levels showed a significant reduction in IL-6 and IL-1β levels in the research group post-treatment compared to baseline (P<0.05). Specifically, the IL-6 levels decreased from (20.87±5.03) pg/mL to (14.72±4.22) pg/mL, and IL-1β decreased from (13.34±3.16) pg/mL to (7.26±1.84) pg/mL. However, the reduction in IL-10 levels, which decreased from (8.84±1.55) pg/mL to (5.24±1.36) pg/mL, was not statistically significant (P>0.05). The control group also showed reduced cytokine levels, but the changes were less pronounced and did not reach statistical significance compared to the research group (P>0.05 for IL-6, IL- 10, and IL-1β levels; Figure 2).

### Psychological status was better in the research group after the treatment

**Figure 3:**
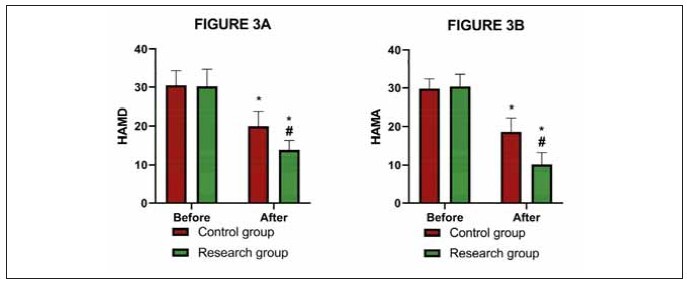
Comparison of A) HAMD and B) HAMA. * denotes P<0.05 compared with before treatment, # denotes P<0.05 compared with control group. Hamilton Depression Scale, HAMD; Hamilton Anxiety Scale, HAMA.

The evaluation results of psychological status revealed that the HAMD and HAMA scores in the research group were (13.91±2.47), (10.20±3.10), respectively, lower than the pre-treatment levels and those of the control group (P<0.05, Figure 3).

### There was no difference in safety between the two groups

According to statistics, the incidence of adverse reactions in the treatment process of the research and control groups was 11.11% and 14.81%, respectively, with no statistical difference between them (P>0.05, Table 2).

**Table 2:** Comparison of adverse effects.

Groups (n=54)	Respiratory depression	Nausea and vomiting	Constipation	Fever	Total Incidence
Control	2 (3.70)	3 (5.56)	2 (3.70)	1 (1.85)	14.81
Research	1 (1.85)	2 (3.70)	2 (3.70)	1 (1.85)	11.11
χ^2^					0.328
P					0.567

### Comparison of delivery outcomes

By comparison, we found no significant intergroup difference in the incidence of premature delivery, polyhydramnios, stillbirth, and fetal malformation (P>0.05); however, the incidence of cesarean section, postpartum haemorrhage, and neonatal asphyxia was higher in the research group than in the control group (P<0.05, Table 3).

**Table 3:** Comparison of delivery outcomes.

Groups (n=54)	Control	Research	χ^2^	P
Premature labour	10 (18.52)	7 (12.96)	0.628	0.428
Cesarean section	22 (40.74)	10 (18.52)	6.395	0.011
Excessive amniotic fluid	5 (9.26)	3 (5.56)	0.54	0.462
Postpartum hemorrhage	13 (24.07)	5 (9.26)	4.267	0.039
Stillbirth	2 (3.70)	0 (0.00)	2.038	0.153
Fetal malformation	1 (1.85)	0 (0.00)	1.009	0.315
Neonatal asphyxia	6 (11.11)	0 (0.00)	6.353	0.012
Macrosomia	4 (7.41)	2 (3.70)	0.706	0.401

### Treatment satisfaction was higher in the research group

Finally, the survey results of patients’ satisfaction with treatment showed that the overall satisfaction of the research group was 94.44%, and that of the control group was 81.48%. The inter-group comparison revealed higher treatment satisfaction in the research group (P<0.05, Table 4).

**Table 4:** Comparison of treatment satisfaction.

Groups (n=54)	Very satisfied	Satisfactory	Needs Improvement	Not satisfied	Total satisfaction
Control	17 (31.48)	27 (50.00)	7 (12.96)	3 (5.56)	81.48
Research	29 (53.70)	22 (40.74)	3 (5.56)	0 (0.00)	94.44
χ^2^					4.285
P					0.038

## Discussion

In recent years, the incidence of PIH is increasing year by year. PIH can cause blood glucose and lipid metabolism disorders, damage vascular endothelial cells, and trigger systemic arteriole spasms [13]. It also damages vascular endothelial cells and increases glomerular permeability [14]. In the case of vasospasms, the blood circulation and blood supply to the placenta will be affected, resulting in reduced enzymatic activity in the placenta and leading to adverse pregnancy outcomes such as fetal growth restriction and neonatal asphyxia [15]. Therefore, a safe and effective treatment scheme for PIH is of great significance to protect the health of pregnant women and their newborns. In this study, we found that MS combined with personalised dietary guidance can effectively improve the hemodynamics of PIH patients, which is significant for the future clinical treatment of PIH.

First, comparing patients’ hemodynamics, it was found that SBP and DBP decreased significantly in both groups after treatment, which is estimated to be related to the positive personalised dietary guidance. We believe that the diet management of PIH can prevent and treat diseases and promote physical rehabilitation. Based on ensuring the necessary nutrition during pregnancy, the poor diet structure of pregnant women with PIH can be corrected, and the total calories can be calculated according to the dynamic monitoring of biochemical indicators, standard body mass, height, and exercise at each stage to modify the diet plan [16]. In addition, dietary guidance rationalises the dietary structure to avoid the effects of over-nutrition or malnutrition on fetal growth and development, thus maintaining the patient’s normal BP and avoiding the increase of other risk events due to persistent hypertension. A study by Marshall NE et al. [17], also emphasised that ensuring the healthy nutritional status of pregnant women through hard management can help reduce the risk of adverse birth outcomes, supporting our view. However, we observed lower BP, PV, and EI in the research group versus the control group after treatment in the intergroup comparison, demonstrating that personalised dietary guidance can further improve the hemodynamics of PIH patients. Pharmacological studies have confirmed that MS can reduce BP quickly by inhibiting acetylcholine release and endothelin synthesis and reducing angiotensin release. At the same time, magnesium ions can effectively anaesthetise the nerve centre, inhibit motor nerve fibre impulses, and dilate blood vessels [18]. In previous studies, the therapeutic effect of MS on PIH has also been verified many times [19] [20], which can support the results of this study.

In addition to improving hemodynamics, our study also explored the effects of MS combined with personalised dietary guidance on inflammatory cytokines, particularly IL-6, IL-10, and IL-1β. The results revealed that the research group showed a significant reduction in the pro-inflammatory cytokines IL-6 and IL-1β post-treatment, which may contribute to the observed improvements in BP and vascular function. Elevated levels of IL-6 and IL-1β are known to be associated with vascular dysfunction and endothelial damage in PIH, which could explain the beneficial effects of reducing these cytokines [21]. Interestingly, while IL-10, an anti-inflammatory cytokine, also decreased, the reduction was not statistically significant. This suggests that while the intervention effectively modulates pro-inflammatory pathways, its impact on anti-inflammatory responses might require further investigation. The reduction in IL-6 and IL-1β levels aligns with the hypothesis that MS, combined with dietary management, can improve hemodynamic parameters and help regulate the immune system, which is crucial in managing the inflammatory aspects of PIH [22] [23]. The excellent anti-inflammatory effects of MS have also been studied in previous studies [24] [25]. In an animal study on late pregnancy by Khatib N et al., they found that MS use inhibited maternal inflammatory responses, oxidative stress and activation of apoptosis, thereby ameliorating brain damage in newborn animals [26]. These findings underscore the importance of targeting inflammation as part of a comprehensive approach to PIH management.

We found lower incidence rates of cesarean section, postpartum haemorrhage, and neonatal asphyxia in the research group compared to the control group in the investigation of delivery outcomes, indicating that MS combined with personalised dietary guidance is of great significance in improving the safety of childbirth for pregnant women. Fetal growth consumes many maternal proteins, calcium, iron, vitamins, folic acid and trace elements, causing anaemia and malnutrition in pregnant women [27]. MS can maintain the normal function of vascular endothelial cells, inhibit the stimulation of vascular endothelium by neuromuscular sensitivity, and supplement vitamins to resist lipid oxidation, thereby preventing and controlling the occurrence of PIH [28]. Meanwhile, the study of Iravani K et al. [29] pointed out that MS can supplement calcium to suppress parathyroid hormone secretion, intervene in the formation of cyclic adenosine monophosphate, reduce cell membrane permeability, and contract blood vessels to achieve the goal of lowering BP, reduce cell membrane permeability, and contract blood vessels to achieve the goal of lowering BP.

Finally, the research group had lower HAMD and HAMA scores and higher treatment satisfaction after treatment. It can be seen that MS combined with personalised dietary guidance can not only ensure the nutritional intake of pregnant women and improve their disease defence ability and immunity but also make them feel the care of others, which helps improve their enthusiasm for treatment. In addition, there is no difference in the incidence of adverse reactions between the two groups, confirming the high clinical safety of MS combined with personalised dietary guidance. However, He W et al. [30] mentioned in their study that MS reduces the incidence of adverse reactions in infants with epileptic spasticity syndrome, which is inconsistent with our findings. It may be due to the accidental statistical analysis caused by the small number of cases in this study, and further confirmation is needed. In addition, the difference between the study population of He W et al. [30], who studied newborns, and our study population of pregnant women may also contribute to the different results.

On the other hand, the follow-up time investigation of the present study did not allow the assessment of the long-term prognosis of pregnant women and newborns. At the same time, we need to add more objective clinical indicators (e.g., coagulation, liver function, renal function, etc.) to more comprehensively assess the full impact of MS and personalised dietary guidance on PIH. In the future, we will conduct more in-depth and comprehensive studies and analyses to address the above limitations.

## Conclusion

The combination of MS and personalised dietary guidance significantly improved hemodynamics and reduced inflammation in pregnant women with PIH, thereby protecting the health of PIH patients and newborns.

### Availability of data and materials

The data supporting this study’s findings are available from the corresponding author upon reasonable request.

### Conflict of interest statement

All the authors declare that they have no conflict of interest in this work.
